# Dancers entrain more effectively than non-dancers to another actor’s movements

**DOI:** 10.3389/fnhum.2014.00800

**Published:** 2014-10-08

**Authors:** Auriel Washburn, Mariana DeMarco, Simon de Vries, Kris Ariyabuddhiphongs, R. C. Schmidt, Michael J. Richardson, Michael A. Riley

**Affiliations:** ^1^Department of Psychology, Center for Cognition, Action and Perception, University of CincinnatiCincinnati, OH, USA; ^2^Center for Human Movement Sciences, University of GroningenGroningen, Netherlands; ^3^Department of Psychology, College of the Holy CrossWorcester, MA, USA

**Keywords:** dance, visual coordination, entrainment, interpersonal coordination, multiscale analysis

## Abstract

For many everyday sensorimotor tasks, trained dancers have been found to exhibit distinct and sometimes superior (more stable or robust) patterns of behavior compared to non-dancers. Past research has demonstrated that experts in fields requiring specialized physical training and behavioral control exhibit superior interpersonal coordination capabilities for expertise-related tasks. To date, however, no published studies have compared dancers’ abilities to coordinate their movements with the movements of another individual—i.e., during a so-called visual-motor interpersonal coordination task. The current study was designed to investigate whether trained dancers would be better able to coordinate with a partner performing short sequences of dance-like movements than non-dancers. Movement time series were recorded for individual dancers and non-dancers asked to synchronize with a confederate during three different movement sequences characterized by distinct dance styles (i.e., dance team routine, contemporary ballet, mixed style) without hearing any auditory signals or music. A diverse range of linear and non-linear analyses (i.e., cross-correlation, cross-recurrence quantification analysis, and cross-wavelet analysis) provided converging measures of coordination across multiple time scales. While overall levels of interpersonal coordination were influenced by differences in movement sequence for both groups, dancers consistently displayed higher levels of coordination with the confederate at both short and long time scales. These findings demonstrate that the visual-motor coordination capabilities of trained dancers allow them to better synchronize with other individuals performing dance-like movements than non-dancers. Further investigation of similar tasks may help to increase the understanding of visual-motor entrainment in general, as well as provide insight into the effects of focused training on visual-motor and interpersonal coordination.

## INTRODUCTION

Human behavior is constantly shaped by the properties, constraints, and affordances of the environment (Gibson, 1986), including the movements of environmental objects and other individuals (e.g., Chartrand and Bargh, 1999; Buekers et al., 2000; Lopresti-Goodman et al., 2008; Richardson et al., 2010). Accordingly, the limb and body movements of actors often become naturally synchronized and coordinated with periodic occurrences in the environment when they are coupled to them via visual (e.g., Dijkstra et al., 1994a,b; Buekers et al., 2000), auditory (e.g., Repp and Penel, 2004; Repp, 2006; Stoffregen et al., 2009), or haptic (e.g., Jeka et al., 1998) information. Evidence indicates that these abilities to engage in multimodal entrainment to environmental rhythms (Phillips-Silver and Trainor, 2005) and to detect cross-modal rhythmic asynchronies (Hannon, 2008) are present early in infancy, revealing a propensity for attunement of human perception and action to such external events.

Of particular relevance to the current study is a large body of research within the field of visual-motor coordination which has demonstrated that actor-environment coordination is governed by dynamical processes of entrainment (e.g., Kelso et al., 1990; Wimmers et al., 1992; Schmidt and Turvey, 1994; Byblow et al., 1995; Wilson et al., 2005) and can be modeled as systems of coupled oscillators (e.g., Haken et al., 1985; Schmidt and Richardson, 2008). This dynamical systems account of actor-environment and interpersonal coordination can be understood as fitting within a broader understanding of behavioral entrainment. More specifically, behavioral entrainment can emerge between an actor and an environmental event or rhythm, or between two or more interacting actors when three critical conditions are met: (1) actors are able to perceive (detect) rhythmic events or behaviors within the environment; (2) actors are producing rhythmic or semi-rhythmic behaviors; and (3) actors are able to adjust behavioral performance based on the perception of the environmental events or behaviors observed (Phillips-Silver et al., 2010). Given these conditions, entrainment can arise spontaneously or can occur as a function of the actor’s intent (Schmidt and Richardson, 2008; Knoblich et al., 2011). Indeed, evidence for both intentional and unintentional visual-motor coordination between an actor and the environment has been observed both in the coordination of postural sway to environmental movements (Dijkstra et al., 1994a,b; Giese et al., 1996; Oullier et al., 2002) and in the rhythmic entrainment of limb or pendulum movements to an oscillating stimulus (e.g., Wimmers et al., 1992; Schmidt and Turvey, 1994; Amazeen et al., 1995; Liao and Jagacinski, 2000; Russell and Sternad, 2001; Kilner et al., 2007; Richardson et al., 2007b; Schmidt et al., 2007; Washburn et al., 2014).

The observation of spontaneous rhythmic limb coordination between two individuals who can see each other’s movements provides further evidence for the natural occurrence of interpersonal entrainment (e.g., Schmidt and O’Brien, 1997; Richardson et al., 2005, 2007a). In comparison to the uni-directional coupling often present in actor-environment coordination, the bi-directional coupling inherent to interpersonal coordination results in an increased sensitivity to situational elements known to affect the stability of visual-motor coordination. As a result, interpersonal coordination is often characterized by less stable and sometimes more relative or intermittent coordination compared to intra-personal or agent-environment coordination (Richardson et al., 2007a; Schmidt and Richardson, 2008). A substantial amount of research has been dedicated to determining what factors have the greatest influence on the occurrence and stability of rhythmic visual and interpersonal coordination (see Schmidt and Richardson, 2008, for a review). For instance, enhancing the informational coupling between the movements of co-actors (Richardson et al., 2007a) or having individuals perform movements that have a comfortable and/or more similar natural frequency (Schmidt and O’Brien, 1997; Richardson et al., 2005, 2007a; Lopresti-Goodman et al., 2008), is known to increase the occurrence and stability of interpersonal rhythmic coordination.

It seems likely that being trained in dance, or any other discipline which often requires high levels of behavioral synchrony, may result in an increased ability to achieve stable, visually mediated interpersonal coordination. While there has not yet been any published research investigating this possibility for dancers (for unpublished work see Issartel et al., 2007; Issartel, 2008), it has been found that expert improvisational actors and musicians show better precision and performance during an improvisatory interpersonal movement mirroring than novices (Noy et al., 2011), and that trained martial artists show better synchronization during interpersonal coordination of a sword swinging task than those who were unfamiliar with the behavior (Schmidt et al., 2011). Additionally, research involving trained dancers has shown that they achieve higher levels of interpersonal synchrony during rhythmic sway in the context of haptic coupling to another individual than non-dancers (Sofianidis et al., 2012a). A number of distinct physical characteristics associated with dance training likely influence such interpersonal movement coordination. For instance, dancers demonstrate better proprioception (Jola et al., 2011; Kiefer et al., 2013), have significantly faster long-latency neuromuscular responses (Simmons, 2005), show more consistent muscle activation (Simmons, 2005), and display stronger interlimb coupling (Sofianidis et al., 2012b) than non-dancers. Dancers also exhibit lower variability of leg rotation during the performance of dance-like movements (Sofianidis et al., 2012b), increased intrapersonal coordinative stability between hip and ankle joint rotations (Kiefer et al., 2011), better postural control (Mouchnino et al., 1992; Golomer et al., 1997; Rein et al., 2011), and dynamically distinct postural sway patterns (Schmit et al., 2005) compared to non-dancers. Trained dancers also exhibit expertise effects in the form of higher levels of synchronization with familiar movements than non-familiar movements (Honisch et al., 2009), and they are skilled at discriminating movements they are most adept at performing (Calvo-Merino et al., 2010).

Dance performance often requires both actor-environment and interpersonal behavioral coordination, as an individual must synchronize with musical events as well as with the movements of others. The interpersonal coordination observed in this context falls within a category of entrainment previously referred to as *social entrainment* or *behavioral synchrony*, and is characterized by one actor’s responsiveness to other actors’ rhythmic behaviors (e.g., Phillips-Silver et al., 2010; Miles et al., 2011). The current study was designed to determine whether dance training is associated with improved visual-motor coordinative abilities, focusing specifically on a social entrainment task. Given the apparent propensity for individuals to entrain their movements to rhythmic beats or music (Phillips-Silver and Trainor, 2005, 2007; Repp, 2005) in such a way that might interact with interpersonal visual-motor coordination, we chose to examine such coordinative abilities in the absence of external auditory stimuli (i.e., counts or music). Dancers and non-dancers were asked to coordinate with a confederate as they performed sequences of dance-like movements. Three distinct movement sequences were choreographed for use in the study, each characterized by a different style (i.e., dance team routine, contemporary ballet, mixed style). This allowed us to evaluate the degree of visual-motor interpersonal coordination achieved by dancers compared to non-dancers across a variety of movement types. In addition, each sequence of steps was composed of several phrases, each metrically defined by eight *counts*, which provided a nested structure of rhythmic movement organization within a phrase. Accordingly, we were able to evaluate the stability of interpersonal coordination for dancers and non-dancers as a function of the different hierarchically nested subcomponents of the dance sequence (i.e., whole phrase, ^1^/_2_ phrase, ^1^/_4_ phrase, and ^1^/_8_ phrase = count-to-count). That is, we were able to evaluate the synchronization structure at the shorter moment-to-moment time scale (i.e., ^1^/_8_ phrase, ^1^/_4_ phrase) and at the longer time scales of the expressive movement sequences (i.e., ^1^/_2_phrase, phrase), both of which were expected to play an important role in shaping the responsive behavior of participants. We hypothesized that dancers would more effectively entrain to the confederate’s movements than would non-dancers, and that the stability of the coordination exhibited by dancers compared to non-dancers would be greater at all the subcomponent time scales of the dance sequences.

## MATERIALS AND METHODS

### PARTICIPANTS

Seventy undergraduate students from the University of Cincinnati participated in the experiment. They ranged in age from 18 to 35 years. Thirty-five participants (16 female, 19 male) did not have formal dancer training, while the other thirty-five participants (31 female, 4 male) were dancers who had at least 5 years of formal dance training, and were either dance majors at the College Conservatory of Music at the University of Cincinnati, members of the University of Cincinnati Dance Team, or members of the University of Cincinnati Cheer Team. The dancer participants had experience in multiple dance styles including ballet, modern, and hip hop. The variability of the dancers’ backgrounds did not permit a systematic test of the effects of training in different dance disciplines. The experiment was approved by the University of Cincinnati Institutional Review Board. All participants provided informed consent.

### PROCEDURE AND DESIGN

Each participant was asked to stand in the center of the room diagonally behind the confederate (1 m behind and 2 m to right), and facing the same direction as the confederate. A Microsoft Kinect for Windows sensor (Microsoft Corp., Redmond, WA, USA) was placed on the floor, ∼2 m in front of the confederate and 0.67 m to the right of the confederate’s starting position. Movement data were collected by the Kinect at a sampling rate of 10 Hz.

**FIGURE 1:**
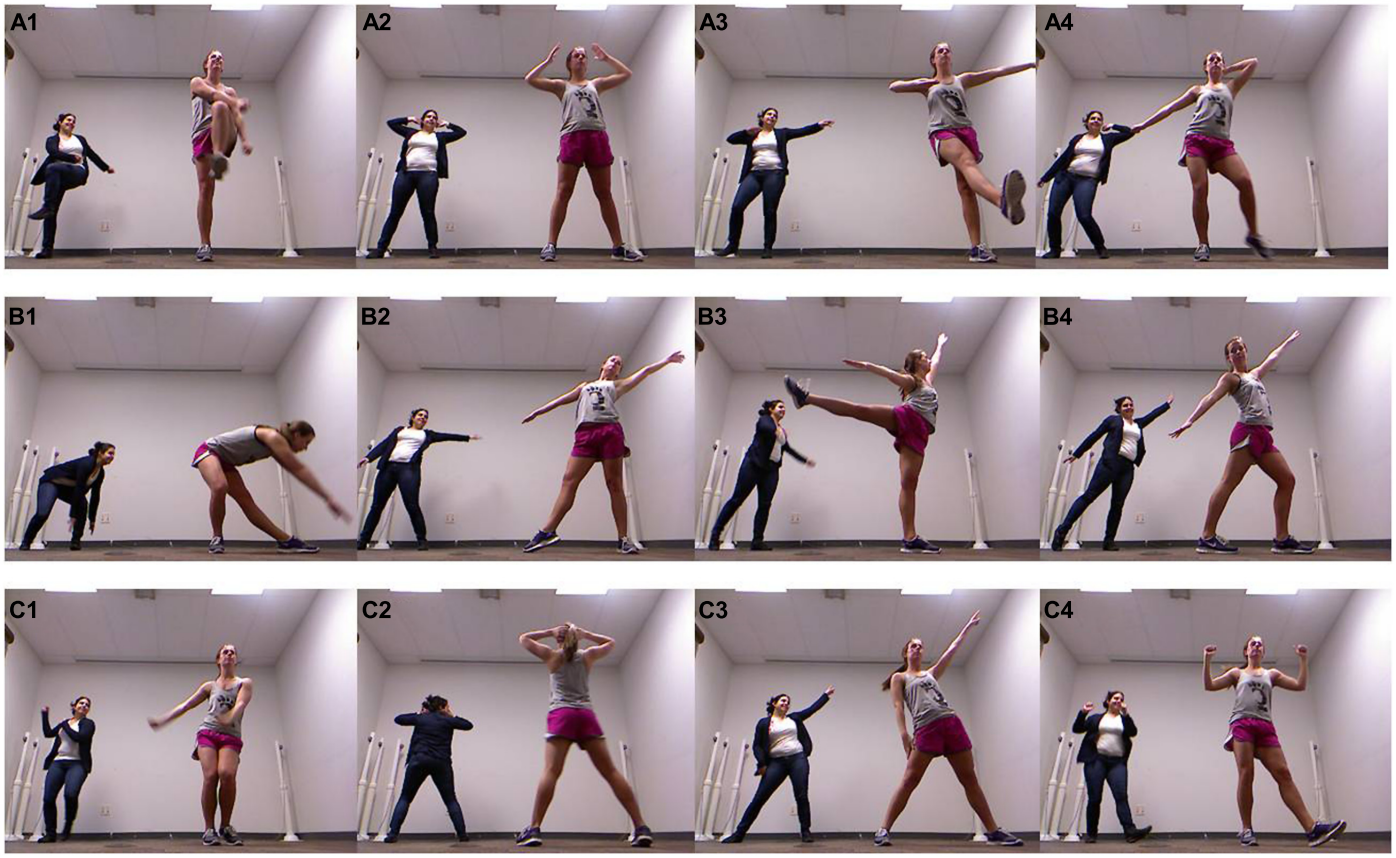
**Example movements from Sequence 1 **(A1–A4)**, Sequence 2 **(B1–B4)**, and Sequence 3 **(C1–C4)**.** For all images the confederate is seen on the right and a non-dancer participant is seen on the left.

Three distinct sequences of dance-like movements were choreographed for use in this study. These sequences differed in terms of both the style of dance movement performed and the difficulty of the moves/dances steps involved. The first and easiest of these sequences was made up of steps commonly observed in dance team routines (**Figure 1, A1–A4**). Most movements were repeated in a symmetrical fashion so that they were performed with the right side of the body, directly followed by the left, although at times contralateral arm and leg movements were employed (e.g., right arm and left leg used simultaneously). The second movement sequence was made up of steps influenced by a contemporary, lyrical ballet style (**Figure 1, B1–B4**). It was intended to be more difficult than the first sequence, but was also choreographed to flow seamlessly from step to step throughout the combination, and consisted mostly of whole body movements, with cohesion between the head, arms, and legs. There was no repetition of movements from one side of the body to the other in this sequence. The third movement sequence was choreographed to be the most difficult sequence and was a compilation of different steps that would not normally be used together (**Figure 1, C1–C4**). Commonly recognized dance moves (e.g., the “disco”) were used, a turn was included, and movements were not left-right symmetrical. As in Sequence 2, there was no repetition of movements from one side of the body to the other in this sequence. All sequences were choreographed using eight count phrases, with each sequence comprising a different number of phrases. Each sequence took ∼60 s to perform. The same confederate dancer performed these sequences each time, so that every trial for each participant involved the coordination of the participant with the same confederate. Sequence presentation was organized into blocks of three, with each sequence presented once in a randomized order. All participants experienced three of these blocks, for a total of nine trials. The confederate, therefore, performed each sequence a total of three times per participant, or 210 times in total.

### DATA PROCESSING

**FIGURE 2:**
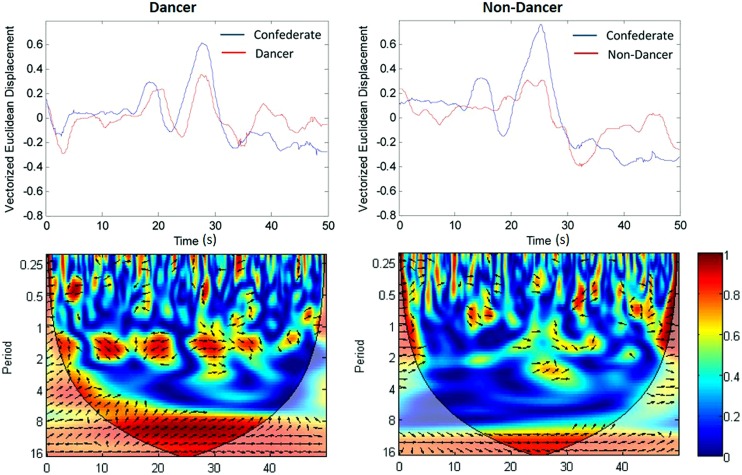
**Movement vector time series **(top)** and cross-wavelet coherence **(bottom)** for a dancer participant coordinated with the confederate **(left)** and a non-dancer participant coordinated with the confederate **(right)**.** Both time series are from performances of Sequence 1. The length of the time series is 50 s (x-axis). The full movement phrase, phrase, phrase, and ^1^/_8_ phrase time scales were 13.25, 6.63, 3.31, and 1.66 s (y-axis periods for cross-wavelet coherence). Coherence magnitude and relative phase at a given time scale and a point in time is denoted by color and the orientation of the arrow (pointing right: inphase; left: antiphase; down: confederate leading by 90°), respectively. The average of these values at a given time scale were extracted from these plots to perform the time scale coherence and relative phase analyses.

The last 50 s of each trial were used for analysis to eliminate transients that occurred at the beginning of each trial. Continuous 3-D position data were obtained for 20 different points of the each of the participant’s and confederate’s bodies (head, center shoulder, right shoulder, left shoulder, right elbow, left elbow, right wrist, left wrist, right hand, left hand, spine, center hip, right hip, left hip, right knee, left knee, right ankle, left ankle, right foot, and left foot) using the Microsoft Kinect sensor. These data were collapsed into overall movement displacement vectors for each trial using the Matlab function “norm”, which computes the spectral norm of a matrix (Mathworks, Natick, MA, USA). The resulting vectors were time series for which each data point corresponded to the overall Euclidean displacement calculated per sample over the 20 body locations and three movement planes. Vectors were zero-centered prior to subsequent analyses (**Figure 2**).

### MEASURES AND ANALYSES

We conducted a set of analyses to comprehensively index interpersonal coordination between the participants and the confederate. Linear cross-correlation analysis was used to provide a measure of overall synchrony between participant and confederate movements for each experimental trial. Cross-wavelet spectral analysis was used to examine the coordinative relationship between participant and confederate movements in further detail by resolving the coordination at the different time scales corresponding to the hierarchically nested subcomponents (time scales) of the dance sequence. Cross-recurrence quantification analysis (CRQA) was used to quantify the shared non-linear structure of the participant and confederate time series with a focus on the maximum line length (MAXLINE) occurring on a cross-recurrence plot (CRP) of participant and confederate behaviors, which quantifies the stability of coordinative relationships between two time series. Although a detailed tutorial of each method employed in the present study is beyond the scope of this paper, in the following subsections we provide a brief introduction to each analysis method and include key references that provide a more thorough description.

#### Cross-correlation

The time series for the participant movements and confederate movements were compared in terms of the cross-correlation coefficient, *x*corr(*h*) (using the Matlab function “xcorr,” Mathworks, Natick, MA, USA), which represents the normalized cross-correlation function of the participant and confederate time series taken at a time lag of *h* samples of the participant with respect to the confederate (see Rabiner and Gold, 1975, for more details about cross-correlation). For each trial, the value of the cross-correlation coefficient between the two time series was calculated for each of a range of time lags up to 5 s, which was deemed a sufficient time span for capturing the overall synchrony of the dance sequences. The values for the maximum cross-correlation coefficient and the time lag at which that maximum occurred were taken to be representative of the overall coordinative relationship between the participant and confederate for a given trial. Trials in which either the maximum cross-correlation coefficient or the time lag was greater than three standard deviations away from the condition (i.e., participant × movement sequence) mean were classified as outliers, and condition means for each participant were calculated without these values. This resulted in the removal of nine trials (4.29% of trials) for Sequence 1, 10 trials (4.76% of trials) from Sequence 2, and six trials (2.86% of trials) from Sequence 3 (overall a total of 3.97% of trials were removed). Average participant cross-correlation coefficient values for each condition were standardized using a Fisher-z transform before statistical analyses were performed.

#### Cross-wavelet spectral analysis

Cross-wavelet analysis is accomplished through spectral decomposition of each time series, and subsequent examination of the degree and pattern of synchronization at each of a selection of component signal frequencies (see Grinsted et al., 2004; Issartel et al., 2006, for a more detailed introduction). More specifically, it evaluates the cross-spectrum of two time series across time, and hence can uncover how the time-localized coherence and relative phase differ at a number of frequency ranges (time scales; **Figure 2**).

**FIGURE 3:**
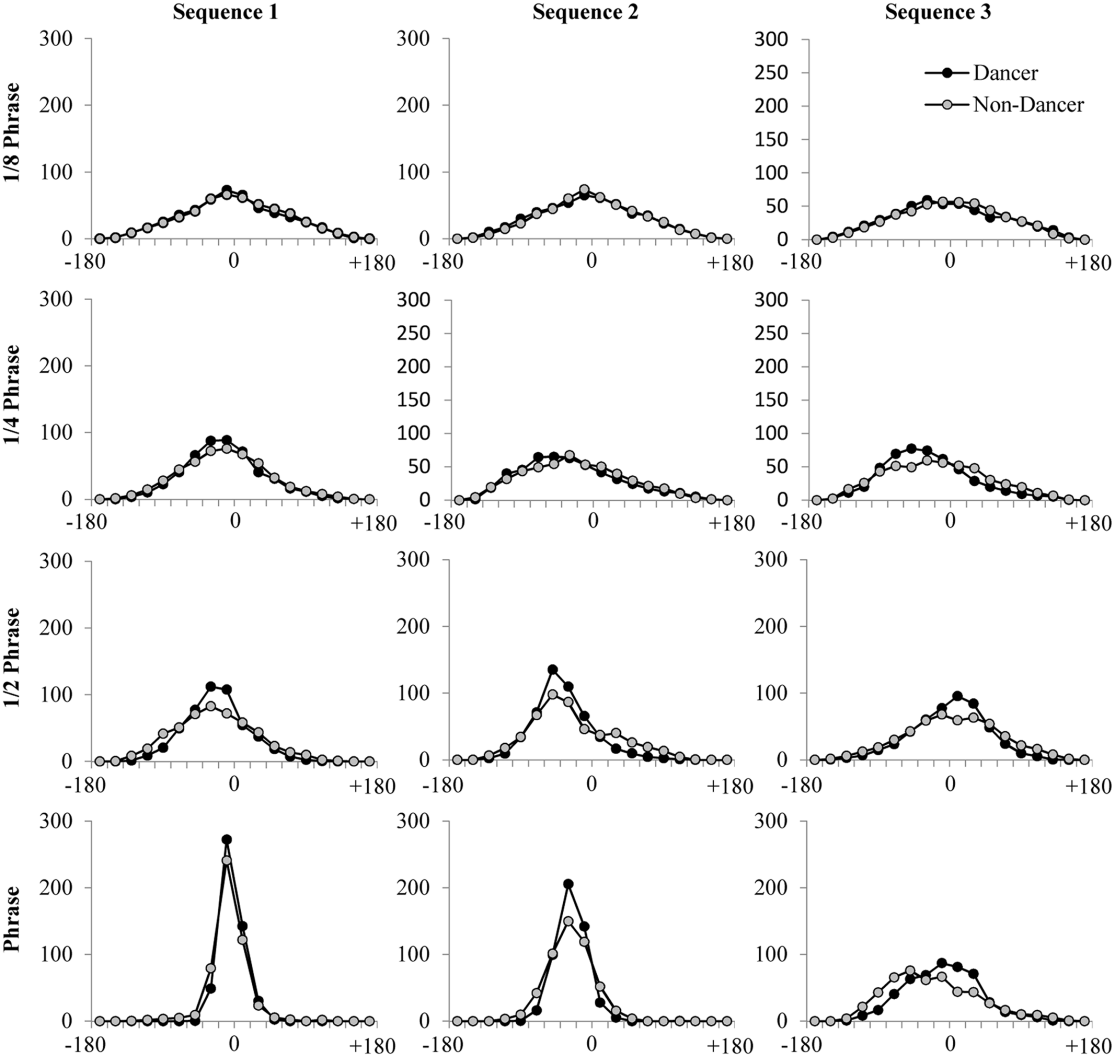
**Average DRP plots for each of the four time scales evaluated using cross-wavelet spectral analysis, for each of the three movement sequences performed**.

In the present study, we used a Morlet wavelet of order 8 to evaluate coherence and relative phase at four distinct time scales. As noted by Schmidt et al. (2012), spectral decomposition of movement time series often reveals behavioral rhythms at nested time scales specific to the task being performed. Here, the spectral peak frequencies examined were associated with the four characteristic time scales of the movement sequences: The whole phrase, the ^1^/_2_ phrase, the ^1^/_4_ phrase, and the ^1^/_8_ phrase (note that ^1^/_8_ of a phrase is approximately equivalent to one count within a phrase). The characteristic frequency for a single phrase was calculated as the inverse of the mean period of movement behavior across experimental trials. This frequency was determined separately for each of the three sequences (Sequence 1: 13.25 s; Sequence 2: 13.22 s; Sequence 3: 14.36 s) and was then employed to define frequency bands for each of the four time scales of interest. These frequency bands included a frequency range ±20% of the frequency corresponding to the phrase time scale (i.e., single, ^1^/_2_, ^1^/_4_, and ^1^/_8_ phrase). For each of these four frequency bands, we then calculated the average bidirectional weighted coherence—a weighted average measure of the correlation of the two time series (where here correlation refers to the extent to which the two time series “share” spectral power at the same frequencies) on a scale from 0 to 1—and the average distribution of relative phase angles (DRP) that occurred between the participant and confederate movement time series, binned into eighteen 20° bins from -180 to 180°. The DRP for each trial and frequency band (**Figure 3**) provides information about how often participant and confederate movements exhibited specific relative phase angle relationships between -180 and 180°. Peaks in the DRP therefore provide both an indication of the temporal relationship between behaviors as well as the stability of that relationship (Schmidt and O’Brien, 1997; Richardson et al., 2007a). The average participant coherence values for each condition and frequency band were standardized using a Fisher-z transform before statistical analyses were performed.

#### Cross-recurrence quantification analysis

Cross-recurrence quantification analysis is an extension of RQA and determines the presence and duration of overlap between the dynamics of two time series by quantifying the regularity, predictability, and stability of two concurrent behavioral performances in reconstructed phase space. Phase space reconstruction is an intermediate step in CRQA. It involves “unfolding” the dynamics of the measured, 1-dimensional time series into a higher-dimensional space. This is done because the measured time series potentially reflects the influences of a larger number of variables that determine the system’s behavior. The influences of those variables are projected onto the 1-dimensional time series that was measured, and this projection from a higher-dimensional space where the system “lives” onto the single axis of the measured variable introduces distortions, much like a 2-dimensional map of the 3-dimensional earth contains distortions. These distortions are apparent as the trajectory of the system overlapping or crossing with itself. Phase space reconstruction pulls apart these false overlaps by embedding the time series in a higher-dimensional space using surrogate dimensions to stand in place of the original system variables. It has been proven (Takens, 1981) that time-delayed copies of the measured time series are adequate to serve as these surrogate dimensions, and the resulting reconstructed phase space is related to the true phase space by smooth, differentiable transforms, which means that the dynamics of the original system are preserved in the reconstructed phase space.

Cross-recurrence quantification analysis requires a number of initial analyses in order to determine several required parameter settings (i.e., reconstruction delay or “lag” and embedding dimension which provide the basis for phase-space reconstruction of the attractor dynamics, as well as a radius parameter described below) for each time series being examined. The following paragraphs briefly describe some of these parameters and how they were selected in the present study. For a more detailed introduction to CRQA and these parameter selection processes see Shockley et al. (2002), Webber and Zbilut (2005, 2007), Marwan et al. (2007), Richardson et al. (2007b), and Webber (2012).

Average mutual information (AMI), a measure of the degree to which the behavior of a time series at one point in time provides knowledge about the behavior of the time series at some other time point (i.e., a non-linear, information theory based variation on the more familiar autocorrelation function), was used here to establish the appropriate delay for phase-space reconstruction (see Abarbanel et al., 1993). Fraser and Swinney (1986) previously identified the time lag at which the first local minimum (*T_m_*) of the AMI function occurs as an appropriate choice for this value as it provides the best estimate of orthogonality with respect to the potential addition of more dimensions within a reconstructed phase space (orthogonality is desired so that the surrogate dimensions used in phase space reconstruction contribute a maximum amount of new information about the underlying system dynamics). Here, we determined *T_m_* for each trial to select the delay.

In order to find an appropriate embedding dimension for the reconstruction of attractor dynamics, the false nearest neighbors (FNN) algorithm was used (see Kennel et al., 1992). The idea behind this process is to unfold the time series in a proxy space of an increasing number of dimensions using the time-delayed (i.e., by *T_m_*, the delay value identified using the AMI analysis) copies of the original time series as the additional dimensions, each time assessing whether apparent crossings of the trajectory with itself are an artifact of being projected within too few dimensions, until none of these “false neighbors” (i.e., data points which overlap or are in close proximity due to projection errors) remain. Similar to the identification of an appropriate reconstruction delay from AMI, FNN analysis was conducted individually for each time series.

After a reconstructed attractor for each behavioral time series was defined using the selected time delay and embedding dimension, CRQA proceeds by then identifying the extent to which two time series (i.e., for the confederate and a participant) share locations or overlap in the reconstructed phase space. “Overlap” means that the two trajectories visited the same region of the phase space within a tolerance defined using a threshold fixed Euclidean distance between points; this radius parameter, as it is termed, was also established as part of the preliminary analyses. Selection of a radius is conducted so that the chance of detected false recurrences is minimized, while ensuring that true recurrences are not missed. Typically, one seeks a radius that ensures that the number of recurrent points within in recurrence plot is between 0.5 and 5% (Shockley et al., 2002; Marwan et al., 2007; Richardson et al., 2007b).

In the current study, preliminary examination of time series allowed us to establish that an embedding dimension of 6 and radius of 30% of the mean distance between points in the reconstructed phase space were characteristic of all trials. Differences in movement dynamics between the three movement sequences used in the current study did appear to affect the characteristic delay parameter for a time series. As such, delay parameters were selected individually for each sequence. For Sequence 1 the preliminary analyses indicated a delay of 18 samples, for Sequence 2 a delay of 20, and for Sequence 3 a delay of 16.

Using the pre-established parameters of embedding dimension, radius, and reconstruction delay, several measures can be obtained from CRQA and can be used to describe different aspects of the structural complexity of concurrent behaviors. For the purposes of the current study, we focused on the length of the longest line of recurrent points on a CRP (MAXLINE), as this captures the longest shared trajectory between the time series and has been shown to be a measure of coupling strength and coordinative stability (Marwan, 2003; Webber and Zbilut, 2005; Richardson et al., 2007b, 2008).

## RESULTS

### CROSS-CORRELATION

**FIGURE 4:**
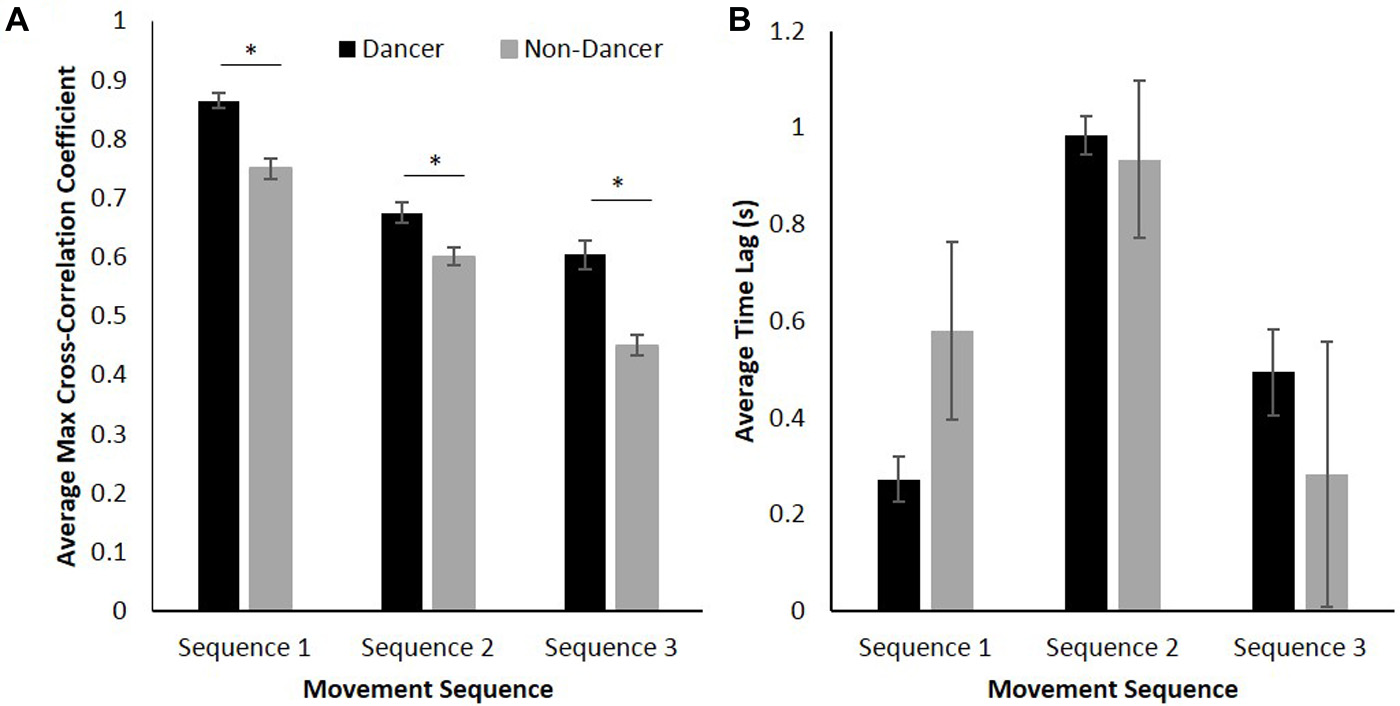
**(A)** Average maximum cross-correlation coefficients for dancer and non-dancer participants, and **(B)** average time lag between dancers coordinated with the confederate and non-dancers coordinated with the confederate. Error bars show standard error. ^∗^ *p* < 0.05.

As can be seen from an inspection of **Figure 4A**, the cross-correlation coefficient magnitude for dancers was consistently greater than that observed for non-dancers. Additionally, for both dancers and non-dancers the cross-correlation coefficients between their movements and those of the confederate were much greater for Sequence 1 compared to both Sequence 2 and 3. Confirming that these differences were significant, a 2 (group: dancer vs. non-dancer) × 3 (movement sequence: 1, 2, or 3) analysis of variance (ANOVA) for the maximum cross-correlation coefficients between participant and confederate movements revealed a significant interaction between group and movement sequence, *F*(2,136) = 6.88, *p* < 0.01, $\eta_{p}^{2}$ = 0.09, as well as significant main effects of both group, *F*(1,68) = 47.40, *p* < 0.001, $\eta_{p}^{2}$ = 0.41, and movement sequence, *F*(2,136) = 199.26, *p* < 0.001, $\eta_{p}^{2}$ = 0.75.

Verifying the expectation that dancers would exhibit greater levels of coordination than non-dancers for all sequences, a simple-effects analysis of group demonstrated significant differences in maximum cross-correlation coefficients between dancers and non-dancers for Sequence 1 [*F*(1,68) = 35.91, *p* < 0.001, $\eta_{p}^{2}$ = 0.35], Sequence 2 [*F*(1,68) = 11.43, *p* < 0.01, $\eta_{p}^{2}$ = 0.14], and Sequence 3 [*F*(1,68) = 27.10, *p* < 0.001, $\eta_{p}^{2}$ = 0.29]. Consistent with the prediction that the level of coordination observed would be higher for Sequence 1 compared to Sequence 2 and 3, a simple-effects analysis of sequence revealed a significant effect for both dancers, *F*(2,68) = 95.64, *p* < 0.001, $\eta_{p}^{2}$ = 0.74, and non-dancers, *F*(2,68) = 119.25, *p* < 0.001, $\eta_{p}^{2}$ = 0.78, with Fisher’s LSD *post hoc* comparisons demonstrating significant differences between all pairs of movement sequences for both groups (*p*s < 0.05). Note that this latter analysis not only reveals that the degree of coordination was greater for Sequence 1 compared to both Sequence 2 and 3, but is also consistent with our expectation that Sequence 2 would be easier than Sequence 3 (i.e., Sequence 1 was easiest; Sequence 3 was hardest).

Finally, a 2 (group: dancer vs. non-dancer) × 3 (movement sequence: 1, 2, or 3) ANOVA was conducted for the value of the time lag at which the maximum cross-correlation occurred between participant to confederate movement. Although there was no significant difference in the associated lag between dancers and non-dancers, *F*(1,68) = 0.02, *p* > 0.05, $\eta_{p}^{2}$ = 0.00 (both dancers and non-dancers lagged slightly behind the confederate), the results revealed a significant main effect of movement sequence, *F*(2,136) = 7.48, *p* < 0.01, $\eta_{p}^{2}$ = 0.10 (**Figure 4B**), with Fisher’s LSD *post hoc* comparisons revealing that the time lag was significantly greater for Sequence 2 compared to both Sequence 1 and 3 (*p*s < 0.01).

### CROSS-WAVELET SPECTRAL ANALYSIS

**FIGURE 5:**
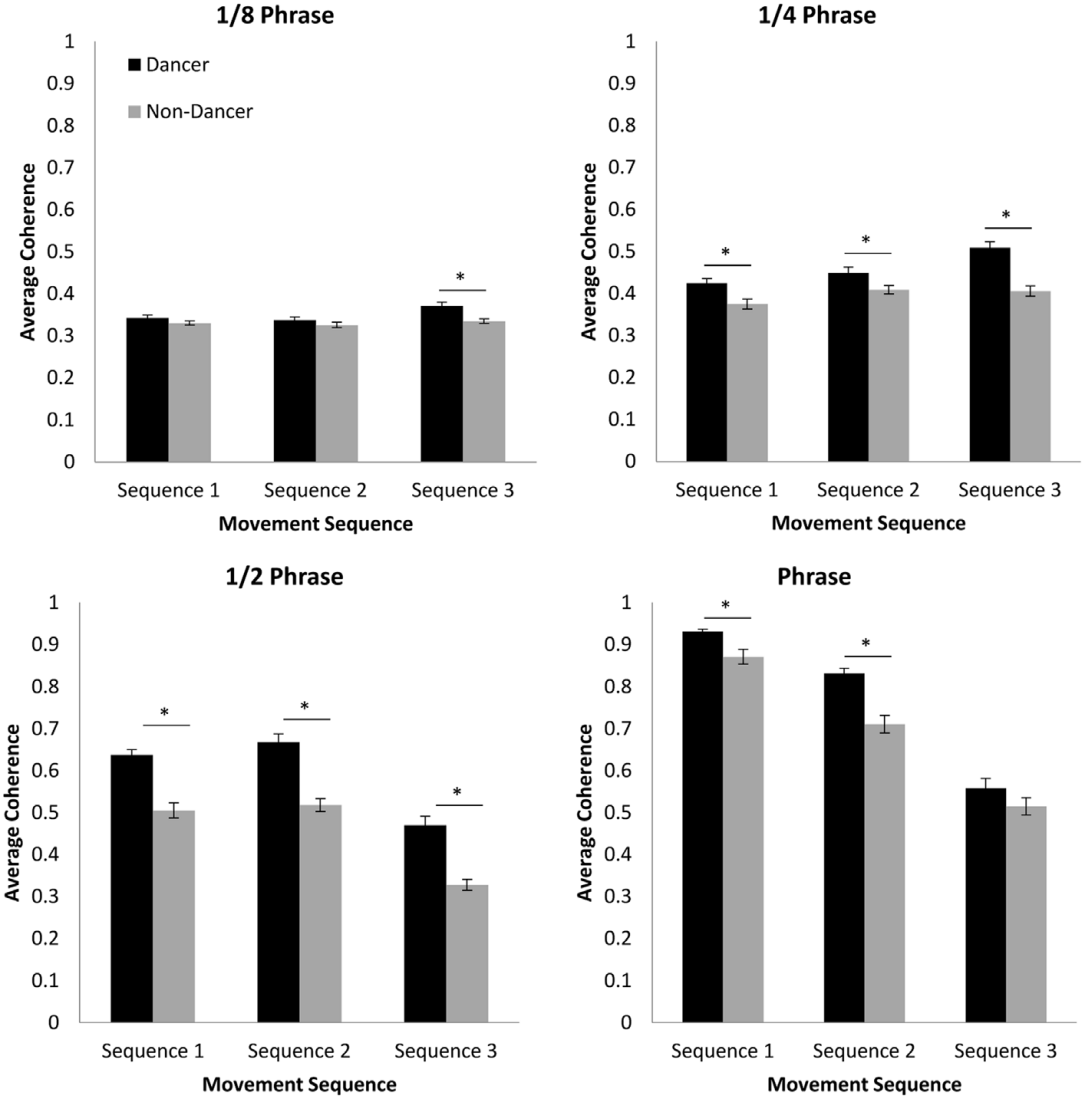
**Average coherence between participant and confederate movements for each of the time scales examined using cross-wavelet spectral analysis.** Error bars show standard error. ^∗^ *p* < 0.05.

In order to address the hypothesis that dancers would be capable of achieving higher levels of coordination than non-dancers at each of the movement phrase subcomponent time scales, separate 2 (group: dancer vs. non-dancer) × 3 (movement sequence: 1, 2, or 3) ANOVAs were conducted to examine the cross-wavelet coherence between participant and confederate movements for each of the four component time scales (^1^/_8_ phrase, ^1^/_4_ phrase, ^1^/_2_ phrase, and full phrase; **Figure 5**).

Although analysis of the ^1^/_8_ phrase time scale resulted in significant main effects of both group, *F*(1,68) = 17.40, *p* < 0.001, $\eta_{p}^{2}$ = 0.20, and movement sequence, *F*(2,136) = 5.48, *p* < 0.01, $\eta_{p}^{2}$ = 0.08, Fisher’s LSD *post hoc* comparisons only found coherence levels to be higher for dancers compared to non-dancers for Sequence 3 (*p* < 0.001). Additionally, although we expected that coherence would be greatest for both participant groups during performance of Sequence 1 and lowest for Sequence 3, levels for Sequence 3 were actually significantly higher than either Sequence 1 or 2 (*p*s < 0.05). The magnitude of this difference was relatively small, however. Furthermore, there was no significant difference between Sequences 1 and 2 (*p* > 0.05). Unlike the cross-correlation results reported above, these results therefore suggest that the difficulty of the dance sequence had almost no influence on the stability of the moment-to-moment coordination for dances and non-dancers, and that dancers and non-dancers were equally capable of moving in a coordinated manner at the time scale of individual movement counts.

For the phrase time scale a significant interaction between variables was observed, *F*(2,136) = 5.91, *p* < 0.01, $\eta_{p}^{2}$ = 0.08, as well as significant main effects of both group, *F*(1,68) = 31.26, *p* < 0.001, $\eta_{p}^{2}$ = 0.32, and movement sequence, *F*(2,136) = 15.89, *p* < 0.001, $\eta_{p}^{2}$ = 0.19. As expected, a simple-effects analysis of group revealed significant differences between dancers and non-dancers for Sequence 1 [*F*(1,68) = 10.32, *p* < 0.01, $\eta_{p}^{2}$ = 0.13], Sequence 2 [*F*(1,68) = 5.89, *p* < 0.05, $\eta_{p}^{2}$ = 0.08], and Sequence 3 [*F*(1,68) = 33.28, *p* < 0.001, $\eta_{p}^{2}$ = 0.33], indicating that at this two-count time scale dancers were better able to coordinate with the confederate than non-dancers. However, although a simple-effects analysis for movement sequence demonstrated that there was a significant effect for dancers, *F*(2,68) = 17.15, *p* < 0.001, $\eta_{p}^{2}$ = 0.34, there was no effect of sequence for non-dancers, *F*(2,68) = 3.14, *p* = 0.05, $\eta_{p}^{2}$ = 0.09. Furthermore, similar to the results for the ^1^/_8_ phrase time scale reported above, Fisher’s LSD *post hoc* comparisons for the simple effect for dancers revealed that coherence was significantly higher for Sequence 3 compared to Sequence 1 and 2 (*p*s < 0.001), and that there was no significant difference between Sequence 1 and 2. Again, this suggests that at the shorter time scales of behavioral coordination, sequence difficulty had only a small influence on the stability of coordination for dancers, and almost no influence on the stability of the coordination for non-dancers.

More consistent with the cross-correlation results, the analysis of coherence for the ^1^/_2_ phrase time scale revealed significant main effects of both group, *F*(1,68) = 77.53, *p* < 0.001, $\eta_{p}^{2}$ = 0.53, and movement sequence, *F*(2,136) = 84.96, *p* < 0.001, $\eta_{p}^{2}$ = 0.56. Consistent with our original expectations, Fisher’s LSD *post hoc* comparisons revealed a significant difference between dancer and non-dancer coherence for all movement sequences (*p* < 0.001). In contrast to the results for the ^1^/_4_ and ^1^/_8_ phrase time scale, the coherence for Sequence 3 at this ^1^/_2_ phrase time scale was found to be significantly lower than that observed for Sequence 1 and 2 (*p*s < 0.001), indicating that the difficulty of Sequence 3 did reduce the degree of behavioral coordination of multi-movement counts for both dancers and non-dancers. There was still no significant difference between Sequence 1 and Sequence 2, however, suggesting that the difficulty of these two sequences may have been similar at the ^1^/_2_ phrase level.

The analysis of coherence for the full phrase time scale revealed a significant interaction between group and sequence, *F*(2,136) = 4.92, *p* < 0.01, $\eta_{p}^{2}$ = 0.07, as well as significant main effects of both group, *F*(1,68) = 34.13, *p* < 0.001, $\eta_{p}^{2}$ = 0.33, and movement sequence, *F*(2,136) = 284.81, *p* < 0.001, $\eta_{p}^{2}$ = 0.81. As was the case for the ^1^/_4_, and ^1^/_2_ phrase time scales, a simple-effects analysis for group revealed that coherence for dancers was significantly greater than for non-dancers for Sequence 1, [*F*(1,68) = 15.01, *p* < 0.001, $\eta_{p}^{2}$ = 0.18] and Sequence 2 [*F*(1,68) = 27.78, *p* < 0.001, $\eta_{p}^{2}$ = 0.29], indicating that dancers were better able to coordinate with the confederate at the scale of the entire phrase for Sequence 1 and 2. Although for the most difficult sequence, Sequence 3, the magnitude of coherence for dancers was also greater than that observed for non-dancers, this difference was not found to be significant [*F*(1,68) = 2.38, *p* > 0.05, $\eta_{p}^{2}$ = 0.03], suggesting that the difficulty of Sequence 3 prevented dancers from better anticipating the longer term structure within each phrase of the sequence compared to non-dancers. The greater difficulty of Sequence 3, was further verified by the simple effects analyses for movement sequence, which revealed a significant effect of sequence for both dancers, *F*(2,68) = 195.30, *p* < 0.001, $\eta_{p}^{2}$ = 0.85, and non-dancers, *F*(2,68) = 104.21, *p* < 0.001, $\eta_{p}^{2}$ = 0.75, with Fisher’s LSD *post hoc* comparisons revealing significant differences between all pairs of movement sequences (*p*s < 0.001). That is, the coherence was lowest for Sequence 3 and highest for Sequence 1 (see **Figure 5**).

Recall that cross-wavelet DRP plots can be used to index the spatial-temporal relationship between the participant and confederate movements at each of the time scales identified, with peaks in the plots providing both an indication of the temporal relative-phase relationship between the participant and confederate, as well as the stability of that relative-phase relationship. As can be seen from an inspection of **Figure 3**, the DRP plots reveal a similar pattern of results as the cross-wavelet coherence, with more stable relative-phase relationships (higher peaks) for dancers than non-dancers. This was especially true for the ^1^/_2_ phrase and full phrase time scales, and for Sequence 1 compared to Sequence 2 and Sequence 3, with Sequence 3 having overall the lowest peaks in the DRP plots. In addition, these plots indicated that at each of the time scales examined there was little difference in the lag between participant and confederate movements for the two participant groups. This is consistent with the lag results from the cross-correlation analysis and suggests that while level of dance skill and sequence difficulty appeared to influence the stability of the participant to confederate coordination, these variables do not influence the degree to which the participant lagged behind the movements of the confederate. On the contrary, the relative-phase lag between participant and confederate remained relatively stable across participant groups and movement sequences.

### CROSS-RECURRENCE QUANTIFICATION ANALYSIS

**FIGURE 6:**
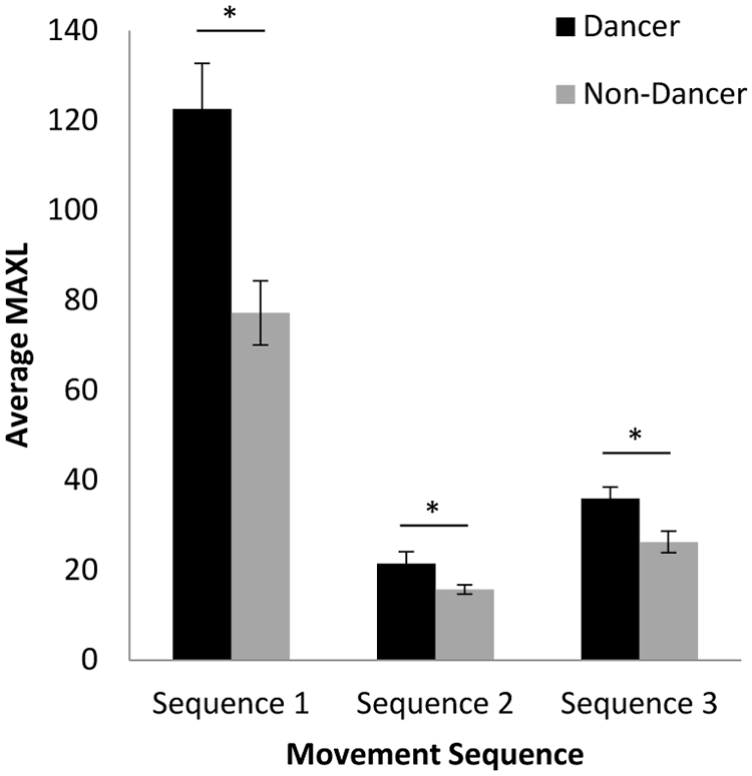
**Average MAXLINE between participant and confederate movements for each movement sequence.** Error bars show standard error. ^∗^ *p* < 0.05.

A 2 (group: dancer vs. non-dancer) × 3 (movement sequence: 1, 2, or 3) ANOVA for the MAXLINE of participant and confederate behaviors revealed a significant interaction between the variables, *F*(2,136) = 10.42, *p* < 0.001, $\eta_{p}^{2}$ = 0.13, as well as significant main effects of both group, *F*(1,68) = 18.66, *p* < 0.001, $\eta_{p}^{2}$ = 0.22, and movement sequence, *F*(2,136) = 145.58, *p* < 0.001, $\eta_{p}^{2}$ = 0.68. As can be seen from inspection of **Figure 6**, the dancer compared to non-dancer MAXLINE results largely mirror the cross-correlation and cross-wavelet coherence analysis for the ^1^/_2_ phrase and full phrase time scales, with MAXLINE being consistently greater for dancers than non-dancers. Accordingly, a simple-effects analysis for group showed significant differences between dancers and non-dancers for Sequence 1 [*F*(1,68) = 14.72, *p* < 0.001, $\eta_{p}^{2}$ = 0.18], Sequence 2 [*F*(1,68) = 4.54, *p* < 0.05, $\eta_{p}^{2}$ = 0.06], and Sequence 3 [*F*(1,68) = 7.90, *p* < 0.01, $\eta_{p}^{2}$ = 0.10]. With respect to the different sequences, MAXLINE was much greater for Sequence 1 than for Sequence 2 or 3 for both dancer and non-dancer participants. Indeed, a simple-effects analysis for sequence revealed a significant effect for both dancers, *F*(2,68) = 87.07, *p* < 0.001, $\eta_{p}^{2}$ = 0.72, and non-dancers, *F*(2,68) = 59.50, *p* < 0.001, $\eta_{p}^{2}$ = 0.64, with Fisher’s LSD *post hoc* comparisons indicating significant differences between all pairs of movement sequences (*p*s < 0.001). Somewhat similar to the ^1^/_8_ and ^1^/_4_ phrase time scale results for cross-wavelet coherence, however, MAXLINE for Sequence 3 was greater than that observed for Sequence 2, which may be a result of CRQA capturing the cross-timescale stability of the coordination. That is, for Sequences 2 and 3, MAXLINE appears to also be picking up the higher levels of short-time scale coordination that were observed at the ^1^/_8_ and ^1^/_4_ phrase time scales for Sequence 3 in the cross-wavelet coherence analysis. It should be noted that the same pattern of results was observed across several CRQA measures, but we have chosen to focus on MAXLINE here as it uniquely addresses the stability of coordination between behaviors with respect to the other coordination analyses used.

## DISCUSSION

Past studies have demonstrated that dance training is associated with many distinct movement-related characteristics and capabilities. These include, but are not limited to, higher levels of interpersonal postural sway coordination in the context of haptic coupling (Sofianidis et al., 2012a), stronger interlimb coupling (Sofianidis et al., 2012b), increased intrapersonal postural (hip-ankle) coordination stability (Kiefer et al., 2011), and better postural control (Mouchnino et al., 1992; Golomer et al., 1997; Rein et al., 2011). The purpose of the present study was to determine if dance training is associated with higher levels of interpersonal, social entrainment in the context of visual-motor coupling. This was evaluated by asking both dancers and non-dancers to synchronize with another actor performing dance-like movements. Results indicate that dancers were better overall at coordinating than non-dancers in this context, with cross-correlation analysis, cross-wavelet spectral analysis and CRQA each providing unique information about the distinctions between dancer and non-dancer entrainment behavior.

The cross-correlation analysis allowed us to assess the overall matching of participant and confederate behaviors across relatively short time scales (within 5 s, or across 1–3 movement counts) in order to determine the degree of local coordination achieved between the participants and the confederate. The maximum cross-correlation coefficients derived from this analysis revealed that dancers were consistently better synchronized with confederate movements than non-dancers, while the associated lag measure indicated no differences between dancers and non-dancers in terms of the temporal delay between participants and the confederate.

The use of cross-wavelet spectral analysis allowed us to gain more information about the entrainment behaviors of dancers and non-dancers across a range of specific phrase subcomponent time scales (see Schmidt et al., 2012), each of which was functionally relevant to the movement sequence coordination task used (i.e., full dance phrase, ^1^/_2_ phrase, ^1^/_4_ phrase, and ^1^/_8_ phrase). Results for the cross-wavelet measure of coherence at the full phrase time scale were largely consistent with the cross-correlation coefficient results in that dancers demonstrated higher levels of coordination than non-dancers. Additionally, for both measures the level of coordination exhibited by both participant groups was highest for Sequence 1 and lowest for Sequence 3. It is important to note here, however, that in contrast to the cross-correlation results, there was no significant difference in coherence between dancers and non-dancers for Sequence 3. Given that this sequence was choreographed using movements that would not typically be found together, we would not necessarily expect to see the same effect of training on the ability for participants to entrain to longer time scale movement gestures as was observed for Sequences 1 and 2. In fact, dance training may have even been a disadvantage in this context. Higher levels of coordination for dancers compared to non-dancers were also seen across movement sequences at the ^1^/_2_ phrase time scale. At this level, behavioral coordination for both groups of participants was lowest for Sequence 3, consistent with the implications of the full phrase time scale results. In other words, it was harder for participants to coordinate with more long-term movement structures for Sequence 3 than either Sequence 1 or 2.

In contrast to the full phrase time scale results, however, there was no difference between coherence levels for Sequence 1 and Sequence 2 for either group of participants, indicating that the difficulty of coordinating with ^1^/_2_ phrase level structures may have been similar for these two sequences. At the ^1^/_4_ phrase time scale, dancers still consistently displayed greater coherence than non-dancers. However, while dancer coordination was sensitive to differences between Sequence 3 and the other two movement sequences, non-dancer coordination was not, indicating that sequence difficulty had much less of an effect on the coordination achieved at this shorter time scale. Differences in the level of coordination produced by dancers vs. non-dancers were much lower at the ^1^/_8_ phrase time scale than any of the other time scales evaluated, with a significant effect of training only observed for Sequence 3. It seems likely that at this time scale, while the overall amount of movement may be similar between participant and confederate, close behavioral synchronization may be relatively uncommon. Interestingly, for both groups of participants the highest levels of coherence at this short time scale were observed during performance of Sequence 3, with no differences observed between Sequence 1 and 2. It therefore appears that dance sequence difficulty did not generally have a substantial effect on coordination at this short time scale. However, for somewhat disjointed movement sequences, such as Sequence 3, participants may better entrain to events on shorter timescales as entrainment to the organization of the larger phrase is harder to achieve.

Peaks in the DRP plots created based on cross-wavelet analysis also provided an opportunity to examine the phase relation between participant and confederate movements at each of the time scales identified (see Schmidt and O’Brien, 1997; Richardson et al., 2007a). Consistent with the lag measure obtained during cross-correlation analysis, these plots indicated that at each of the time scales examined there was little difference in the lag exhibited by dancer and non-dancer participants. Additionally, higher peaks suggested greater stability of coordination for dancers than non-dancers, especially for the phrase and full ^1^/_2_ phrase time scales.

The implication of these cross-wavelet results, therefore, is that skilled dancers were not necessarily more coordinated to the moment to moment (count-to-count) movements of the confederate than non-dancers, but that skilled dancers were more coordinated with the long term phrase structure (whole phrase, ^1^/_2_ phrase, ^1^/_4_ phrase) of the different sequences than non-dancers. In other words, the differences in coherence and DRP for dancers and non-dancers at the longer time scales suggest a difference in the functional relevance of the whole phrase, ^1^/_2_ phrase, and ^1^/_4_ phrase time scales for dancers compared to non-dancers. That skilled dancers are able to coordinate effectively at both short and long time scales, as opposed to untrained individuals who only appeared to be able to effectively coordinate on a count-to-count basis, indicates that the perception-action processes of skilled dancers may be more “tuned” to the information about sequence structure and upcoming movement possibilities than non-skilled dancers. Hence the psychological reality of the nested dance events might be much more extended in time for dancers compared to non-dancers. This idea is further supported by the finding that increased exposure to contemporary dance sequences is associated with an improved ability to distinguish novel “grammatical” sequences, as defined by regularities across a series of discrete movements, from those that are non-grammatical (Opacic et al., 2009). The current results highlight the utility of analysis techniques like cross-wavelet analysis in order to test such hypotheses and point to a new way of defining dance skill.

Recall that the three movement sequences used in the current study were designed to emphasize different stylistic qualities and to provide varying levels of difficulty. In keeping with our objective of making Sequence 1 the simplest and most repetitive of all sequences, the results of the cross-correlation analysis, as well as the cross-wavelet DRP plots, revealed higher levels of coordination during this sequence than for the other movement sequences. The same was true for cross-wavelet coherence at the ^1^/_2_ and full phrase-level time scales. The cross-wavelet coherence levels were lowest for Sequence 1 at the ^1^/_4_ and ^1^/_8_ phrase time scales, with Sequence 3 having marginally higher levels of coherence. MAXLINE for Sequence 3 was also found to be higher than for Sequence 2. This suggests that for the current task sequence difficulty had little impact on the overall level of coordination and coordinative stability at the shorter time scales examined. Differences in coordination and coordination stability between movement sequences were observed primarily at longer behavioral time scales, indicating that our manipulation of difficulty primarily influenced the ability of participants to entrain to movement gestures and events which spanned a substantial portion of a phrase, or the full phrase itself. Previous research has indicated that individuals are sensitive to the amount of structure displayed in a sequence such that even for dancers who have expertise in both ballet and modern dance recall is better for the more structured sequences found in ballet (Jean et al., 2001). Additionally, dancers display higher levels of synchronization with familiar movements than non-familiar movements (Honisch et al., 2009). It is therefore not surprising that the visual-motor coordination abilities of trained dancers do appear to be affected by characteristics of the discrete movements and the larger structure of a given sequence. Still, dancers are consistently able to maintain superior coordinative abilities in comparison to those who do not have dance training.

During joint dance performance each dancer is necessarily engaged in *social entrainment* (Phillips-Silver et al., 2010) through their responsiveness to other performers, musicians, or even audience members. Within this context an even larger-scale *collective social entrainment*, characterized by a network of connections among individuals (Phillips-Silver et al., 2010), can also emerge. Actor engagement in shared rhythmic timing during joint dance performance (Keller and Rieger, 2009) is likely shaped by both this collective social entrainment and by actor-environment entrainment to musical events at the level of each individual. By employing a movement coordination task in which no auditory stimulus was provided, the current study demonstrated that dancers are capable of more closely entraining to the dance-like movements of another individual, even without the organizing influence of rhythmic auditory events. Evaluation of the results using a variety of analysis methods allowed us to show that the coordinative processes responsible for such entrainment are occurring on multiple, nested time scales. This is consistent with existing theories about the complex nature of social interactions (Newtson, 1993), as well as empirical work that has examined multi-scale interpersonal coordination during conversation (Schmidt et al., 2012).

Previous studies have shown that trained dancers exhibit strong interlimb coupling (Sofianidis et al., 2012b) which may be reflective of specialized movement synergies, demonstrate proficiency at optimizing task constraints in such a way that enables the performance of complex physical tasks (Kiefer et al., 2011), and are more accurate at synchronizing movement dynamics than positions (Honisch et al., 2009). Additionally, research examining visual-motor coordination for individuals trained in other disciplines which require specialized physical training has shown that experts achieve higher levels of interpersonal coordination for tasks related to their area of expertise (Noy et al., 2011; Schmidt et al., 2011). In light of these findings, we propose two possible, non-exclusive explanations for the higher levels of coordination and coordination stability exhibited by dancers in the current study. One possibility is that the kinematic quality of dancer movements may be more similar to that of the dancer confederate due to skill and training, allowing dancers to produce movement trajectories that more closely resemble the confederate’s than the trajectories of non-dancers. Along with the finding that dancers’ physical experience allows them to better discriminate those movements they are more adept at performing (Calvo-Merino et al., 2010), this explanation would suggest that dancers more accurately perceive and match behaviors with which they have more experience. While the current finding that dancers did not display significantly higher levels of coordination for Sequence 3 might appear to contradict this possibility, the atypical organization of behaviors experienced here may be understood as disrupting an individual’s ability to maintain coordination across a series of movements. Another possibility is that dancers are better capable of coupling to and anticipating other actor’s movements, independent of their ability to produce similar movement quality. Either of these possibilities would permit a person trained in dance to create a stronger interpersonal synergy (Riley et al., 2011) with a partner. Further research in this area will help to expand our understanding of what specific behaviors and skills allow dancers to better achieve visual-motor social entrainment and possibly establish a new understanding of dance skill in terms of creating such dynamical interpersonal synergies.

Regardless of the underlying processes that might have supported the improved coordination exhibited by the trained dancers, the current findings have several implications for interpersonal and actor-environment coordination, in general. In addition to the fact that visual-motor coordination between co-actor movements plays a foundational role in the successful completion of many joint-action tasks, it has also been shown that the coordination of movement patterns between two individuals via visual information can increase interpersonal rapport, reduce prejudice, and facilitate social awareness (e.g., Miles et al., 2010, 2011). Increased coordination ability resulting from dance training could therefore improve performance of other tasks and activities in which multiple actors must synchronize or coordinate movements and subsequently lead to increases in social connectivity. It is also possible that they might have positive effects on group cohesion and communication (Dale et al., 2013). Furthermore, the close entrainment of a single individual to ongoing environmental events associated with enhanced behavioral coupling would likely be advantageous and adaptive in a variety of everyday contexts, such as driving on the highway or crossing a busy street during one’s daily commute. Ultimately, the present work provides new insights about the impact of dance training on visual, interpersonal coordination and demonstrates that the understanding of such social entrainment processes is greatly enriched through the use of multi-scale behavioral analysis.

## Conflict of Interest Statement

The authors declare that the research was conducted in the absence of any commercial or financial relationships that could be construed as a potential conflict of interest.
